# Editorial: Return to sport (RTS): identifying injury risk factors, diagnostics and treatment process

**DOI:** 10.3389/fmed.2024.1415900

**Published:** 2024-04-30

**Authors:** Anna Mika, Łukasz Oleksy, Chris J. Bishop

**Affiliations:** ^1^Institute of Clinical Rehabilitation, University of Physical Education in Krakow, Kraków, Poland; ^2^Faculty of Health Sciences, Department of Physiotherapy, Jagiellonian University Medical College, Kraków, Poland; ^3^Oleksy Medical and Sports Sciences, Rzeszów, Poland; ^4^London Sports Institute, Middlesex University, London, United Kingdom

**Keywords:** return to sport, sports medicine, asymmetry, musculoskeletal system, diagnostics methods, rehabilitation, injury prevention

Contemporary sports impose extraordinary physical demands on athletes, thereby concurrently elevating the likelihood of injury occurrence. Accumulated scientific evidence illustrates that previous injuries exert a significant influence on the likelihood of subsequent injuries. Furthermore, this susceptibility escalates with each successive injury encountered. The correlation between prior injury and the heightened risk of subsequent injury can consequently result in damage, not only to the same anatomical structures, but also to surrounding and even unrelated structures. This phenomenon arises from alterations in movement patterns, deteriorating balance, and other functional or psychological impairments (1).

In the realm of sports, non-contact injuries encompass a diverse range of traumas incurred without direct physical interaction with another player or object. These injuries often stem from intrinsic factors such as: biomechanical imbalances, neuromuscular deficiencies, or overuse, alongside extrinsic factors like: environmental conditions or inadequate equipment (2). Understanding the biomechanical and physiological foundations of non-contact injuries is paramount for formulating preventive strategies. These strategies are likely to include: targeted strength and conditioning programs, proper technique training, and adequate rest intervals. Moreover, interventions aimed at addressing modifiable risk factors, such as: biomechanical imbalances or training errors, can help mitigate the incidence of non-contact injuries in sports (3, 4).

This Research Topic includes four manuscripts: three of which are review articles and one brief research report. In the first study Sun and Liu systematically compiled a collection of high-quality prospective studies pertaining to musculoskeletal issues prevalent in modern and contemporary dancers, along with their associated risk factors. The authors calculated sensitivity pooled effect from a random effects model (DerSimonian-Larid) and the fixed effects model (Mantel–Haenszel) was utilized to analyse various risk factors and dance-related injuries. Consequently, the authors proposed a series of scientifically-grounded intervention measures aimed at injury prevention. Specifically, the evidence indicates the correlation between five distinct factors and the likelihood of injury, highlighting previous injury history and high BMI as notable risk factors among dancers. Examination of prevalence rates reveals a widespread occurrence of musculoskeletal injuries within dancers', particularly with a significant prevalence of overuse injuries and those affecting the lower extremities. Consequently, it is recommended that modern and contemporary dancers maintain a healthy body mass proactively utilizing prevention techniques and only resuming dance training after fully recovering from injury, thereby mitigating potential adverse consequences.

In the second study, Gaddi et al. highlighted the absence of a standardized protocol for managing acute ankle sprains, despite their frequent occurrence in sport practice. In this article, the authors conducted an evaluation of the most prevalent treatments for acute ankle injuries, analyzing the level of evidence presented in previous systematic reviews. This umbrella review revealed that non-surgical interventions prove effective in managing acute ankle sprains, with functional treatment emerging as a preferable option over immobilization. Additionally, both paracetamol and opioids demonstrate comparable efficacy to NSAIDs in pain reduction, offering an alternative treatment avenue. Furthermore, manipulative and exercise therapies show promise, particularly in the initial recovery phase, aiding in the prevention of reinjury and facilitating the restoration of dorsiflexion.

In the third study, Xu et al. represents the inaugural bibliometric and visualization analysis of Patellofemoral Pain Syndrome (PFPS) research spanning the past 23 years, offering multifaceted insights into the field. This comprehensive examination provides a fresh perspective, facilitating a rapid comprehension of PFPS research trends. In the realm of sports medicine, PFPS stands as a prevalent condition, with the surge in sports competitions correlating with a heightened incidence of sports-related injuries. Despite the escalating volumes of research relating to PFPS, a notable dearth in bibliometric analyses addressing this condition persists. The current state of research underscores a substantial reservoir of untapped potential within the PFPS domain. Emphasis should be placed on areas such as the clinical efficacy of combined hip and knee strengthening interventions for PFPS management, elucidation of lower limb kinematics and biomechanics, gait retraining methodologies, identification of risk factors, and formulation of clinical practice guidelines.

In the fourth study Legnani et al. focused on diagnosing limb assymetries post ACL reconstruction when establising criteria for guiding RTS (return to sport). They indicated that the utilization of objective tests for risk assessment in sports injuries confers an advantage over subjective methods owing to its measurability. The authors underlined, that at the 6-month mark post-ACL reconstruction the majority of patients exhibited unsatisfactory knee functional performance, whereas a notably higher percentage met the RTS criteria 1 year post-surgery. Their findings indicate that, the recovery of muscle coordination appears to commence around the 6-month post-surgery mark, with explosive leg power and neuromuscular control showing tendencies to approach their pre-surgical levels rather than experiencing substantial improvements 1 year later. The results reinforce the idea that the timeframe for resuming cutting and pivoting sports should be extended beyond 6 months, given the persistence of limb asymmetries during this period. Furthermore, the treatment process involving an interdisciplinary team holds particular significance in sports. Understanding mutual competencies and specialization in specific actions is crucial, as better outcomes can be achieved when collaboration is based on the amalgamation of skills and knowledge from multiple specialists, albeit specialized in narrower disciplines.

In conclusion, the articles presented in this Research Topic underscore the intricate relationship between sports, injuries, and the imperative need for preventive measures and informed management strategies. From dancers to athletes recovering from ACL reconstruction, each study illuminates distinct facets of injury occurrence, treatment modalities, and rehabilitation protocols. These findings not only contribute to the scientific discourse surrounding sports medicine but also offer tangible insights for coaches, athletes, and healthcare practitioners alike. Moving forward, continued research efforts and interdisciplinary collaboration will be pivotal in advancing our understanding of sports injuries and enhancing the wellbeing and performance of athletes across diverse sporting disciplines. By embracing evidence-based interventions and fostering a holistic approach to athlete care, we can strive toward a future where sports remain not only exhilarating but also safe and sustainable endeavors for all participants.

## Author contributions

AM: Conceptualization, Supervision, Writing – original draft, Writing – review & editing. ŁO: Conceptualization, Supervision, Writing – original draft, Writing – review & editing. CB: Conceptualization, Supervision, Writing – original draft, Writing – review & editing.
